# Clinical-Pathological Correlation of KRAS Mutation Status in Metastatic Colorectal Adenocarcinoma

**DOI:** 10.4021/wjon719w

**Published:** 2013-09-27

**Authors:** Karen Bento Ribeiro, Karoline Bento Ribeiro, Omar Feres, Jose Joaquim Ribeiro da Rocha, Liane Rapatoni, Sergio Britto Garcia, Alfredo Ribeiro Silva, Gleici da Silva Castro Perdona, Hayala Cristina Cavenague de Souza, Saul Isaac Garrido Santillan, Harley Francisco de Oliveira, Daniela Pretti da Cunha Tirapelli, Fernanda Maris Peria

**Affiliations:** a Division of Clinical Oncology, School of Medicine of Ribeirao Preto, University of Sao Paulo (Faculdade de Medicina de Ribeirao Preto, Universidade de Sao Paulo-FMRP-USP), Sao Paulo (SP), Brazil; b Internal Medicine, Federal University of Triangulo Mineiro (Universidade Federal do Triangulo Mineiro - UFTM), Minas Gerais (MG), Brazil; c Department of Surgery and Anatomy, FMRP-USP (SP), Brazil; d Department of Clinical Oncology, FMRF-USP (SP), Brazil; e Pathology, FMRP-USP (SP), Brazil; f Computer Science and Computational Mathematics, Department of Social Medicine, FMRP-USP, Brazil; g Community Health, FMRP-USP, Brazil, BA in Statistics, Federal University of Sao Carlos (Universidade Federal de Sao Carlos - UFSCar), Brazil; h Gastroenterology Surgeon, Sao Jose General and Maternity Hospital (Hospital e Maternidade Sao Jose), Uberaba, MG, Brazil; i Radiation Oncology, FMRP-USP (SP), Radiotherapy Service, FMRP-USP (SP), Brazil; j Department of Surgery and Anatomy, FMRP-USP (SP), Brazil; k Clinical Oncology, FMRP-USP (SP), Clinical Oncology Service, FMRP-USP (SP), Brazil

**Keywords:** Colon neoplasms, Pronto-oncogene proteins p21 (ras), Epidermal growth factor receptor, Tumor biological markers, Survival analysis

## Abstract

**Background:**

KRAS gene mutations play an important role in the carcinogenesis of colorectal tumors. However, studies that have assessed the association between KRAS gene mutation status and disease characteristics report conflicting results. To assess KRAS gene status (mutated or wild-type) and its association with the clinical, epidemiological, and histopathological features of metastatic colorectal adenocarcinoma as well its association with clinical outcomes.

**Methods:**

Cross-sectional descriptive study in which clinical and histopathological data were collected from the medical records of 65 patients diagnosed with metastatic colorectal adenocarcinoma at the Clinical Oncology Service of the Teaching Hospital of the School of Medicine of Ribeirao Preto, University of Sao Paulo (Hospital das Clinicas da Faculdade de Medicina de Ribeirao Preto, Universidade de Sao Paulo -HCFMRP-USP) between 2005 and 2012 and analyzed based on their KRAS gene status.

**Results:**

KRAS gene mutations were found in 49.2% of the tumors, and G/A (25.5%) and Gly12Asp (34.37%) were the most frequent mutations. Among the investigated clinical features (gender, ECOG (Eastern Cooperative Oncology Group), histology, degree of cell differentiation, lymph node ratio, primary tumor site, staging, presence of synchronous metastasis, lung metastasis, and liver metastasis), the association between age less than 65 years with KRAS mutation was statistically significant (P = 0.046). KRAS mutation status did not exhibit a significant correlation with the overall survival of the patients (P = 0.078); however, the cases with KRAS mutation exhibited shorter survival. In the multivariate analysis, synchronous metastasis (P = 0.03) and liver metastasis (P = 0.008) behaved as independent factors of poor prognosis relative to the overall survival of the patients.

**Conclusion:**

The KRAS mutation status did not exhibit prognostic value in the investigated sample. Among the older patients (> 65 years old), wild-type KRAS was more frequently observed compared to mutated KRAS.

## Introduction

Cancer poses a significant public health problem, as it is the cause of approximately 12% of deaths worldwide. Colorectal neoplasms are the third most frequent type of cancer among females and the fourth most common type of cancer among males and cause approximately 610,000 deaths per year worldwide. The number of new cases of colorectal cancer in Brazil was estimated as 14,180 among males and 15,960 among females in 2012. The American Cancer Society (ACS), National Cancer Institute (NCI), Centers for Disease Control and Prevention (CDC), and North American Association of Central Cancer Registries (NAACCR) reported an estimated 1,660,290 new cases of cancer and 580,350 cancer-related deaths in the United States in 2013, including 102,480 new cases of colon cancer, 40,240 new cases of rectal cancer, and 59,830 deaths by colorectal neoplasms. Thus, colorectal cancer is responsible for nearly 10% of cancer-related deaths in the USA, with liver metastases being the main cause of death [1].

Curative treatment is based on primary tumor resection and complementary adjuvant therapy when needed. The systemic treatment of advanced and metastatic disease has made significant advances in recent decades. Initially, only the anti-metabolic chemotherapeutic agent fluorouracil was available, and its use resulted in a mean survival of 12 months. The development of novel cytotoxic agents (irinotecan and oxaliplatin) improved survival (18 to 24 months), symptom relief, and quality of life. The current development of molecular targeted therapies has optimized the therapeutic response rates, increasing the overall survival to approximately 30 months [2-5].

The prognosis of patients with colorectal cancer depends on several criteria, including tumor staging according to the TNM classification (AJCC- American Joint Committee on Cancer /UICC- International Union Against Cancer) as well as histological and molecular characteristics [6]. The presence of obstructive, ulcerated, and perforated tumors, a lymph node ratio (LNR = ratio of the number of lymph nodes exhibiting neoplastic cells to the total number of dissected lymph nodes) higher than 0.16, mucinous histology, neuroendocrine component, signet ring and medullary cells, poorly differentiated or undifferentiated tumors, high cellular proliferation index, and KRAS gene mutation is associated with a high risk of relapse and disease progression [1, 6, 7]. In addition, the presence of metastasis (TNM stage IV), especially in the liver and/or lungs, is associated with an 8% reduction in the five-year survival. Liver and lung metastases are present in 20-70% and 10-20% of patients at the time of diagnosis, respectively [1].

KRAS oncogenic mutations occur in approximately 40% of colorectal tumors. This phenomenon results in a mutated Ras protein that constitutively activates the epidermal growth factor receptor (EGFR) signaling pathway and stimulates cell proliferation and carcinogenesis through the mitogen-activated protein kinase (MAPK) signaling pathway [2, 8-10].

KRAS mutations occur most frequently in codons 12 (80%) and 13 (17%). These mutations correspond to missense mutations (point change of a single nucleotide) and lead to the encoding of a different amino acid and the consequent synthesis of a functional or nonfunctional protein [10].

Clinical studies demonstrate that the presence of KRAS mutations is predictive of resistance to anti-EGFR monoclonal antibodies and is associated with lower disease-free (DFS) and overall (OS) survival [4, 5, 11].

The results of the major studies that have assessed the prognostic value of KRAS mutations are conflicting. Some studies have reported a correlation between the functional ability of mutated Ras protein, specific mutations, predictive value, and clinical outcomes [9, 12-16]. Andreyev et al (2001) conducted the largest study to date (3,439 patients with metastatic colorectal adenocarcinoma) that assessed the relative impact of various KRAS gene mutations. Multivariate analysis indicated that a single glycine-to-valine mutation in codon 12 exhibited a significant effect on DFS (P = 0.004, HR (Hazard ratio): 1.3) and OS (P = 0.008, HR: 1.5). Lievre et al (2008) conducted a study with 89 patients and reported that patients who exhibited mutated KRAS had poorer OS (10.1 versus 14.3 months, P = 0.026) and DFS (10.1 versus 31.4 weeks, P = 0.0001) compared with patients with wild-type KRAS. The KRAS mutation status behaved as an independent prognostic factor of OS (P = 0.0001) and DFS (P = 0.001) in multivariate analyses.

Milano et al (2008) conducted a study with 39 patients and observed an association between KRAS mutation status and tumor stage (52.9% of KRAS-mutated tumors were in stages II to IV versus 23.8% of tumors without the gene mutation, P = 0.035). Tie et al (2011) conducted a study with 100 patients with metastatic colorectal adenocarcinoma and found a significant association between KRAS mutation and lung metastases (62%), regardless of the primary tumor site (colon or rectum) (P = 0.003). In another study conducted at a national center, Ghezzi et al (2011) investigated 37 patients with metastatic colorectal adenocarcinoma and found no correlation between TNM stage and KRAS mutation status.

Li et al (2012) conducted a study with 78 patients with colorectal adenocarcinoma and reported a correlation between KRAS mutation status, liver metastases (P < 0.05), and poor tumor differentiation (P < 0.05). Univariate analysis revealed that KRAS mutation was predictive of poor OS (P = 0.023).

The present study investigated the presence of KRAS mutations (mutated or wild-type) and their association with clinical-epidemiological and histopathological tumor features as well as the clinical outcomes of patients with metastatic colorectal adenocarcinoma.

## Methods

In this descriptive cross-sectional study, clinical and histopathological data were retrospectively collected from medical records and analyzed as a function of KRAS mutation status (mutated or wild-type). Only patients with metastatic colorectal adenocarcinoma assisted at the Clinical Oncology Service of the Teaching Hospital, Medical School of Ribeirao Preto, University of Sao Paulo (Hospital das Clinicas, Faculdade de Medicina de Ribeirao Preto da Universidade de Sao Paulo - HCFMRP-USP) between February and April 2012 who were subjected to primary tumor resection between 2005 and 2011 at the Proctology Service of HCFMRP-USP were included in the study. The study was approved by the Research Ethics Committee of HCFMRP-USP (no. 14277/2011).

Statistical analysis was performed using SPSS version 15 software. The descriptive analyses of the continuous quantitative variables are expressed as the mean, median, absolute frequency, and percentage. Chi-square (χ^2^) and Fisher’s tests were used to analyze the categorical variables. The non-parametric Kaplan-Meier estimator was applied to the analysis of survival, and the log-rank test was used to calculate the differences in the overall survival curves. Multivariate analysis was performed by means of binary logistic regression. The significance level was established as P < 0.05.

## Results

The KRAS mutation status and the clinical and pathological tumor features were assessed in a total of 65 patients (Table 1). The sample comprised a slightly greater number of females (56.9%), and the mean age of the patients was 56.2 years (22 - 84 years old). More than half of the participants (58.5%) exhibited an ECOG (Eastern Cooperative Oncology Group) performance status of 0 at the time of diagnosis. In approximately 56.9% of the cases, the primary tumor was a rectal adenocarcinoma, and 83.1% of patients did not exhibit obstructive and/or perforated acute abdomen at the time of diagnosis.

**Table 1:** Distribution of K-RAS Mutation Status and Clinical-Pathological Tumor Features in Colorectal Cancer Patients

Clinical-pathological features	Total (n = 65)	K-RAS wild-type (n = 33)	K-RAS mutated (n = 32)	P-value
Gender				0.195
Male	28	12	16	
Female	37	21	16	
Age				0.046
> 65 years	44	30	14	
< 65 years	21	3	18	
ECOG				0.495
0	38	17	21	
1	25	15	10	
2	2	1	1	
Histological type				0.256
Adenocarcinoma	50	27	23	
Mucinous	15	6	9	
Cell differentiation				0.221
well	49	27	22	
intermediate	16	6	10	
LNR > 0.16	(60)			0.371
Yes	34	17	17	
No	26	11	15	
Lung metastasis				0.531
Yes	34	16	18	
No	31	17	14	
Liver metastasis				0.663
Yes	43	21	22	
No	22	12	10	
Synchronous metastasis				0.051
Yes	35	14	21	
No	30	19	11	
Primary tumor site				0.914
Colon	28	14	14	
Rectum	37	19	18	
Obstructive and/or perforated acute abdomen				0.273
Yes	11	7	4	
No	54	26	28	
Staging				0.074
II	13	10	3	
III	17	9	8	
IV	35	14	21	
Recurrence				0.067
Yes	56	31	25	
No	9	2	7	

ECOG: Eastern Cooperative Oncology Group; LNR: lymph node ratio.

Approximately 53.8% of the participants were in stage IV of the disease at the time of diagnosis, and the metastases were classified as metachronous (metastases found at least six months after the diagnosis of the primary tumor) in 46.2% of the cases. Most tumors were pure adenocarcinomas (76.9%) and exhibited intermediate differentiation (84.6%). More than 90% of the participants were subjected to lymph node dissection, and 43.1% of these patients had more than 12 lymph nodes dissected. Approximately 56.6% exhibited an LNR above 0.16.

At the end of the follow-up and data analysis, 52.3% of patients exhibited liver metastases, and 66.1% of patients exhibit lung metastases. Disease progression or relapse occurred in 86.2% of the sample population. Mutations in the KRAS gene were identified in 49.2% of the participants.

The clinical and pathological features of the patients were correlated with the KRAS mutation status (Table 1). Age less than 65 years, namely, that of the youngest participants, exhibited a significant correlation with the presence of the mutated gene (P = 0.046).

Molecular analysis was performed relative to the mutations in KRAS codons 12 and 13 in exon 2, including five mutations in the former codon and two in the latter (Table 2). The mutations most frequently observed were as follows: G/A (62.5%), G/T (34.37%), G/C (31.25%), and Gly12Asp (34.37%).

**Table 2:** Distribution of K-RAS Mutation Types in Colorectal Cancer Patients, Point Mutations and Amino Acids Exchanged

Codon	Missense mutation	Amino acid Wild-type	Amino acid mutated	n = 32 (%)
12 (n = 24)	G-A	GGT (Gly)	GAT (Asp)	11 (34.37%)
	G-T	GGT (Gly)	GTT (Val)	6 (18.75%)
	G-T	GGT (Gly)	TGT (Cys)	4 (12.5%)
	G-A	GGT (Gly)	AGT (Ser)	2 (6.25%)
	G-C	GGT (Gly)	GCT (Ala)	1 (3.125%)
13 (n = 8)	G-A	GGC (Gly)	GAC (Asp)	7 (21.87%)
	G-T	GGC (Gly)	GTT (Val)	1 (3.125%)

n: number of patients; G: nucleotide glycine; A: nucleotide alanine; T: nucleotide thymine; C: nucleotide cytosine; Gly: nucleotide glycine; Asp: nucleotide aspartate; Val: nucleotide valine; Ala: nucleotide alanine; Ser: nucleotide serine; Cys: nucleotide cisteine.

No significant correlation was observed between the clinical and pathological features of the patients and KRAS mutation status (G/T and G/A) (gender, P = 0.447; age above 65 years old, P = 0.134; ECOG, P = 0.337; histological type, P = 0.606; degree of cell differentiation, P = 0.409; lymph node affection, P = 0.979; lung metastasis, P = 0.364, liver metastasis, P = 0.510; synchronous metastasis, P = 0.619; obstructive/perforated acute abdomen, P = 0.447; tumor size, P = 0.447; and relapse of disease, P = 0.646).

Binary logistic regression was performed to assess the likelihood of liver metastasis as a function of the primary tumor site (colon or rectum) and KRAS mutation status (mutated or wild-type). Neither the tumor site nor the KRAS mutation status were found to influence the likelihood of liver (P = 0.160) or lung (P = 0.579) metastasis. The primary colon tumors were associated with an increased risk of liver metastasis, and the primary rectum tumors were associated with an increased risk of lung metastasis (Table 3).

**Table 3:** Binary Logistic Regression Analysis and Distribution of the Probabilities of Occurrence of Liver and Lung Metastases According to Primary Site and Tumor KRAS Mutation Status

		Kras wild-type	Kras mutated	P-value
Liver metastasis	colon	P: 0.766581	P: 0.804848	0.160
	rectum	P: 0.540414	P: 0.596230	
Lung metastasis	colon	P: 0.424453	P: 0.504118	0.579
	rectum	P: 0.529350	P: 0.607908	

P: probability.

From the multivariate analysis, only the presence of synchronous metastasis (P = 0.003, RR (relative risk): 2.92) and liver metastasis (P = 0.008, RR: 2.65) independently influenced the survival curve of the patients, whereas the presence of the KRAS gene mutation did not have any significant effect (P = 0.078) (Table 4).

**Table 4:** Multivariate Analysis of Clinical-Pathological Features Associated With Prognostic Value in Colorectal Cancer Patients

Characteristics	Relative Risk	CI (Confidence interval) 95%	P-value
K-RAS mutation	-1.76	-0.9537 - 0.0501	0.078
Cell differentiation,	-0.79	-0.8236 - 0.3492	0.428
Gender	-0.74	-0.6267 - 0.2829	0.459
Age	0.47	-0.0118 - 0.0191	0.642
Primary tumor site	1.16	-0.1708 - 0.6679	0.245
Lymph node involvement	-1.62	-0.8142 - 0.0763	0.104
Liver metastasis	2.65	0.2427 - 1.6257	0.008
Lung metastasis	-1.56	0.7462 - 0.0859	0.120
Synchronous metastasis	2.92	0.2961 - 1.4998	0.003
Histological type	-0.70	-0.6845 - 0.3248	0.485

**Figure 1:**
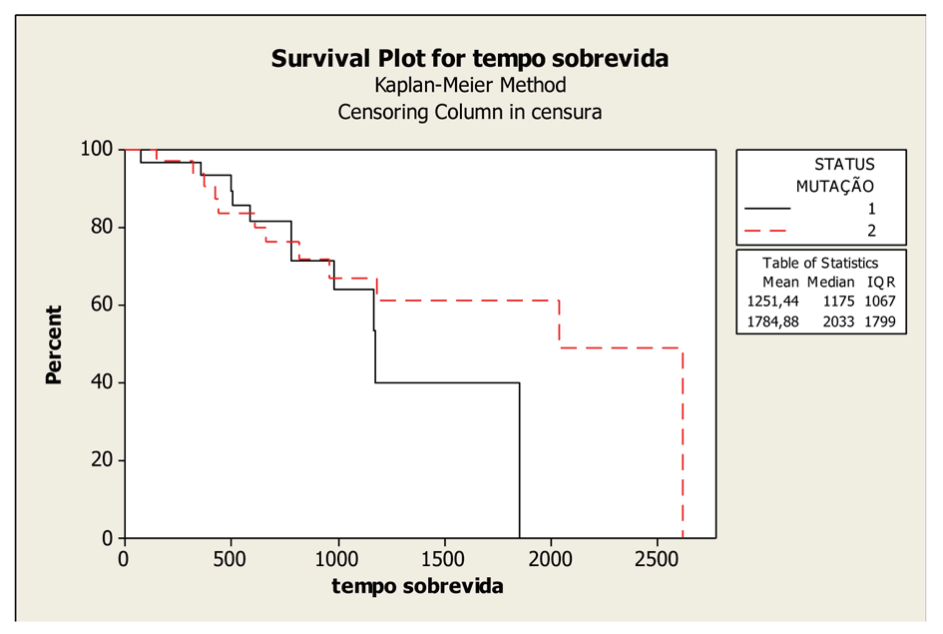
Kaplan-Meier Overall Survival Curves according to KRAS gene status (mutated and wild-type), P-value = 0.407. Legend: K-RAS mutated: 1; K-RAS wild-type: 2.

The mean OS was 58.73 months (45.76 to 71.7 months), with a median of 67 months in the patients with wild-type KRAS; and 41.95 months (32.92 to 50.98 months) with a median of 39 months in the patients with mutated KRAS; and the difference between them was not significant (P = 0.407). In absolute terms, the patients with wild-type KRAS lived longer compared to those with mutated KRAS (Fig. 1).

**Figure 2:**
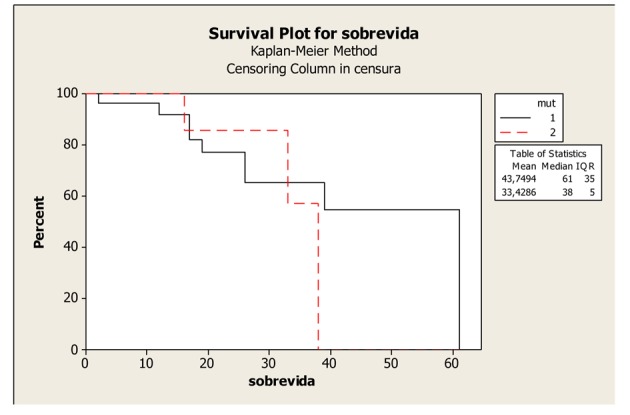
Kaplan-Meier Overall Survival Curves of patients with K-RAS mutated according to mutation in codons 12 and 13, P-value = 0.651. Legend: K-RAS mutated codon 12: 1; K-RAS mutated codon 13: 2.

The mean survival time did not differ as a function of the mutation site, namely, codon 12 or 13 of the KRAS gene. The mean survival was 43.74 months (33.89 to 53.6 months) and the median survival was 61 months in patients with a mutation in codon 12, compared with a mean survival of 33.42 months (26.39 to 40.46 months) and a median survival of 38 months in patients with a mutation in codon 13 (P = 0.651), (Fig. 2).

## Discussion

The incidence and mortality rates of colorectal cancer are high, especially among older adults, and recent research has focused on the identification of predictive and prognostic biomarkers. Within this context, the KRAS gene bears significant predictive value relative to the use of anti-EGFR therapies in colorectal neoplasms. According to the literature, KRAS mutations occur in 35 to 45% of tumors [10], consistent with the results of the present study (49.2%).

The concordance between the KRAS mutation profile of the primary tumor and the profile of the corresponding metastases is high (90%), suggesting that these active somatic mutations are acquired early during carcinogenesis and before metastasis. In the present study, the KRAS status was investigated only in the primary tumor specimens [17-19].

KRAS mutations occur more often in codon 12, where they typically consist of the replacement of glycine by aspartate (G/A-30.6%) followed by the glycine-to-valine mutation (G/T-23.4%), and in codon 13, where they consist of a glycine-to-aspartate replacement (G/A-16.7%) [18]. The results of the present study thus agree with the reports in the literature, as the most frequent replacement found was G/A (62.5%), followed by G/T (34.3%), in codons 12 and 13 of the KRAS gene [20].

The current literature and large historical series discuss the possible correlation between particular nucleotides involved in missense mutations and the clinical and pathological features of the disease. G/C, G/A and G/T replacements might be correlated with the various histological tumor subtypes. Point mutations in codon 12 (nucleotide changes resulting in the formation of the amino acid valine) are associated with the production of mucinous tumors and with lower OS and DFS [20]. G/T mutations result in lower OS and DFS rates [13, 21, 22]. The types of mutations and particular amino acids that are predictive of relapse and metastasis sites have not yet been fully elucidated.

No significant correlation was found between the clinical (gender, ECOG, perforated/obstructive acute abdomen, tumor stage, lung or liver metastasis, primary tumor site) and pathological (histological type, LNR) features and KRAS mutation status. These findings are consistent with the results of other studies [13-15, 21-23]. In the RASCAL study and the study conducted by Rako et al (2012), the frequency of poorly differentiated tumors was lower among the mutated KRAS tumors [13, 23]. In the present study, age less than 65 years (P = 0.046) was significantly correlated with the presence of KRAS mutation, whereas wild-type KRAS was more frequently observed among patients older than 65 years of age. Conversely, the abovementioned studies did not observe any correlation between age and KRAS mutation status, and age was not considered an independent prognostic factor.

The presence of synchronous or metachronous metastasis did not exhibit a significant correlation with the KRAS mutation status. Nevertheless, synchronous metastases were more frequently observed in mutated KRAS tumors, thereby denoting a more aggressive form of the disease (P = 0.051). Some authors reported a significant correlation between the degree of cell differentiation (well and moderately differentiated) and the KRAS mutation status (mutated), suggesting the relevance of the KRAS signaling pathway in cell differentiation [14, 21].

Tie et al conducted a study with 160 patients and found an association between the presence of mutations in codon 13 and non-mucinous histology, lymph node metastases, and Dukes’ stage C/clinical stage III [14]. The mutations in codon 12 were associated with mucinous tumors, thereby suggesting that the mutation might preferentially influence the signal transduction pathway involved in the regulation of mucin production, although the relationship between the mutation and the regulation of cell proliferation has not yet been proven. The univariate analysis revealed that infiltrating tumor growth, absence of peritumoral lymphocyte infiltration, and the presence of lymph node metastases were independent predictors of poor prognosis. From the multivariate analysis, advanced Dukes’ stage, tumors with high aneuploidy, and mutated KRAS codon 13 were the most significant prognostic factors [14].

Some studies have reported an association between the KRAS mutation status and resistance to treatment with anti-EGFR tyrosine-kinase inhibitors (cetuximab and panitumumab). The prognostic value of the KRAS mutation status has not yet been well established in the literature, and the results of studies are conflicting [12-15, 21, 24]. In the RASCAL study, multivariate analysis revealed that tumors with mutated KRAS exhibited a greater risk of relapse (P < 0.001) and death (P = 0.004). The CALGB 89803 group compiled the data from 508 patients with colorectal adenocarcinoma stage III and did not find any prognostic association between KRAS mutation status and relapse (P = 0.89) or OS (P = 0.56) [24].

The OS curves in the present study exhibited a non-significant difference between the patients with wild-type and mutated KRAS (P = 0.407). This fact might be due to the small sample size, which may not have allowed for the identification of a possible association between mutated KRAS and poor prognosis.

Among the patients with mutated KRAS, the survival curves differed as a function of the mutation site, namely, codon 12 or 13. Survival was worse in patients with mutations in codon 13.

In the RASCAL study, which was designed to assess the effect of the phenotypic expression of the KRAS mutation subtypes, only one specific mutation in codon 12 (glycine-to-valine replacement, present in 9% of the colorectal tumors) was associated with poor outcomes (30% increase in relapse and death). These findings might be explained by the existence of various pathways of carcinogenesis resulting from different KRAS mutations (codons 12 and 13), thereby resulting in dissimilar outcomes [8, 13, 21].

In disagreement with the literature, the present study did not identify an association between KRAS mutation status and the primary tumor site (colon or rectum) or the localization of the lung and liver metastases. According to some series, the presence of mutated KRAS is predictive of lung metastases in patients with primary colon tumors [15, 18, 19]. From the regression analysis, the KRAS mutation status (mutated or wild) did not influence the likelihood of liver metastasis (P = 0.673); however, when the colon was the primary site, the likelihood of liver metastasis was greater, whereas the likelihood of lung metastasis was higher in the case of primary rectal tumors.

Local or distant relapse is directly related to the biological behavior of the tumors, whereas doubts exist as to the relevance of the primary tumor mutation profile to the relapse site. The available data remain limited and suggest that KRAS mutations tend to be more prevalent in lung (58%) compared with liver (32%) metastases [19]. Recent data compiled from a large series of studies suggest that KRAS mutations in the primary tumor might be associated with an increased risk of lung and brain metastases but bear no correlation with the risk of liver metastasis. Patients with wild-type KRAS exhibit a decreased risk of lung metastasis compared with liver metastasis [18, 19].

In some case series, multivariate analysis revealed that KRAS mutations, the presence of liver metastasis, and the tumor degree of differentiation are independent predictors of poor prognosis, whereas other series did not find any association between the clinical and pathological tumor features and KRAS mutation status [22, 23, 25]. In the present study, multivariate analysis identified synchronous metastasis and liver metastasis as independent predictors of poor prognosis, whereas the KRAS mutation status did not influence the prognosis of the study population (P = 0.078).

An analysis of the KRAS mutation site as a possible prognostic biomarker was not performed in the present study due to the small sample size. The clinical outcomes of colorectal cancer vary as a function of the location of the mutation in either codon 12 or 13, thereby suggesting that carcinogenesis might follow different pathways depending on the mutation site [13, 15, 20-22].

An analysis of the results indicated different clinical features and outcomes between the colorectal cancer patients with wild-type and mutated KRAS. The literature does not provide evidence on the prognostic and/or predictive relevance of the KRAS mutation. In theory, the predictive value of the KRAS gene relative to the relapse and prognosis of colorectal adenocarcinoma is most likely associated with highly specific mutations involving nucleotide changes distributed along the codons, which result in the production of different amino acids, persistent protein Ras activity, and the amplification of this signaling pathway, even in the absence of effectors and regulators of extracellular stimulation. The degree of GTPase activity and the ability of KRAS to interact with regulators and effectors are suggested to vary as a function of the duration and intensity of the transduction signaling activation, resulting in a more persistent state of Ras protein activation and altering the balance between cell differentiation and proliferation, thereby promoting aggressiveness that may facilitate carcinogenesis.

Studies employing genetic silencing of KRAS in cell culture and experimental models might contribute to a better understanding of this relevant pathway (Ras pathway) of colorectal carcinogenesis, whereas multicenter studies with large samples might elucidate the doubts related to the clinical use of the KRAS mutation status. The search for biomarkers is of paramount importance in the stratification of the risk of relapse and metastasis as well as in the assessment of the response to treatment, allowing for individualized therapies and the clinical and radiological monitoring of high-risk patients.

### Conclusions

Mutated KRAS was observed in approximately 49.2% (32 patients) of metastatic colorectal cancer in a patient sample comprising 65 individuals.

Multivariate analysis revealed that synchronous metastasis (P = 0.003) and liver metastasis (P = 0.008) behaved as independent factors associated with poor survival.

The KRAS mutation status did not behave as a prognostic factor in the study sample (P = 0.0078).
